# Slippage boosted spectral cleaning in a seeded free-electron laser

**DOI:** 10.1038/s41598-019-43061-5

**Published:** 2019-05-06

**Authors:** Chao Feng, Xingtao Wang, Taihe Lan, Meng Zhang, Xuan Li, Junqiang Zhang, Wenyan Zhang, Lie Feng, Xiaoqing Liu, Haixiao Deng, Bo Liu, Dong Wang, Zhentang Zhao

**Affiliations:** Shanghai Advanced Research Institute, CAS, 239 Zhangheng Road, Shanghai, 201204 China

**Keywords:** Ultrafast lasers, Free-electron lasers

## Abstract

The realization of fully coherent light sources at extreme ultraviolet to x-ray region has been a long-standing challenge for laser technologies. While modern single pass free-electron lasers (FELs) hold the ability to produce very intense x-ray radiation on few-femtosecond timescale, the output radiation pulses usually have noisy spectra and limited temporal coherence since the amplification starts from electron noise. A promising way for producing stable transform-limited pulses is based on the harmonic up-conversion techniques with a conventional laser as the seed. However, it is found that the insignificant phase error in the seed laser may be eventually multiplied by the harmonic number, leading to a degradation of the output temporal coherence at x-ray wavelength. Here, we report for the first time on the demonstration of a slippage boosted spectral cleaning technique to mitigate the impact of seed laser induced phase errors and to significantly improve the temporal coherence of a seeded FEL with large phase errors in the seed laser. Experimental results indicate the possibility of generating fully coherent x-ray radiation pulses with this technique.

## Introduction

The recent development of ultrashort optical laser technology has spurred a large variety of applications in science research and information industry due to its superior properties such as fully coherent, high stability and extremely high intensity. When the conventional lasers attempt to working in the extreme ultraviolet (EUV) and x-ray spectral regions, some insurmountable difficulties arise. Different alternative methods have been developed to extend the spectral coverage in optical physics^1–4^. Indeed, the laser-driven sources offer several comparatively inexpensive and widely available options, but practical bottlenecks in high intensity requirements are still encountered.

Free-electron laser (FEL) utilizes free, or un-bound electrons as the gain medium to amplify the initial electromagnetic field. Unlike conventional lasers, the output wavelength, temporal durations and peak brightness of a high-gain FEL are theoretically unlimited, making it a unique and innovative approach for the realization of a tunable, ultra-intense, coherent light source in the EUV and x-ray regions. To date, several EUV/x-ray FEL facilities have been constructed worldwide^5–8^ and have already enabled the observation and control of very fast phenomena at the atomic time scale.

Most of the existing and the under-construction x-ray FEL facilities take advantage of the self-amplified spontaneous emission (SASE) principle^9,10^, which can provide radiation pulses with perfect spatial coherence, high peak power at hundreds of GW level and pulse duration of sub-femtoseconds to hundreds of femtoseconds^11,12^. However, originating from shot noise of the electron beam, the SASE FEL is usually characterized by poor temporal coherence and large fluctuations. One of the major goals for the current development of FELs is to generate laser-like properties in the x-ray range, i.e. radiation pulses with fully temporal coherence, controllable phase and stable power from shot-to-shot.

One possible way to improve the temporal properties is to employ an external coherent laser source as the “seed” to dominate the FEL gain process. In a typical seeded FEL scheme, e.g. coherent harmonic generation (CHG) or high-gain harmonic generation (HGHG)^13^, a seed laser pulse is first sent into a short undulator (called modulator) to introduce an energy modulation into the electron beam. When this electron beam passing through the following magnetic chicane (called dispersion section), electrons with different energy will move relative to one another as they traverse different path length. This longitudinal dispersion converts the sinusoidal energy modulation into density modulation (periodic current peaks). Since the density modulation contains components at high harmonics of the seed, powerful radiation pulse at shorter wavelength can be obtained in the following undulator (called radiator) that resonates at a target harmonic of the seed.

Ideally, it is anticipated that the radiation pulse from a seeded FEL should inherit the properties of the seed laser, e.g. stable and fully coherent, which has been experimentally demonstrated at low harmonics (~10) for HGHG at several FEL facilities^14–18^. Nevertheless, in order to generate coherent radiation at EUV/x-ray wavelength range, the required harmonic number should be on the order of several tens to hundreds, and several challenges and difficulties appear in implementing the HGHG technique. For example, the high harmonic number for a single stage HGHG will be constrained by the beam energy spread; the output temporal coherence will be affected by various energy curvatures in the electron beam^19^. In particular, the initial small phase errors of the seed laser will be amplified during the harmonic up-conversion process and may eventually overwhelm the external seeding source^20,21^ at short wavelengths.

In the EUV region, it has been demonstrated that fully coherent pulses can be achieved by fine tuning the operation parameters of the seed laser and the dispersion section to compensate the combined effects from the beam energy curvature and the chirp developed during the FEL amplification^22,23^. For shorter wavelength, some novel schemes such as echo-enabled harmonic generation (EEHG)^24,25^ and phase-merging enhanced harmonic generation (PEHG)^26,27^ have been developed, and it has been demonstrated that the spectral bandwidth of EEHG is less sensitive to the nonlinear energy chirp of the electron^28–30^. These theoretical and experimental results pave the way for seeding a soft x-ray FEL directly from an ultraviolet laser. But it is still a problem on how to mitigate the effect of the initial high order and random phase errors in the seed laser on the FEL output coherence.

The longitudinal properties of the FEL pulse are largely determined by the distribution of the microbunching in the electron beam. It has been investigated that undulator sections resonant at sub-harmonics of the FEL wavelength can be used to enhance the FEL slippage, which provides an *in situ* method for communicating phase information over larger portions of the electron beam and improving the temporal coherence of a SASE FEL^31,32^. Similarly, we have proposed the idea of using a modulator resonant at sub-harmonics of the seed for slippage-boosting purpose in a seeded FEL^33^. In this scheme, the modulator resonates at an odd sub-harmonic of the seed laser:${\lambda }_{m}=m{\lambda }_{s}$, *m* = *3*, *5*, *7*, *…*,^34^ which can enlarge the slippage length by *m* times while still keeping the FEL interaction in the small-gain regime in the modulator. For a normal HGHG with *m* = 1, the microbunching distribution is determined by the phase of the seed laser, and results in a phase error amplification for high harmonics. However, for the proposed scheme with sub-harmonic modulation, the imperfection of the seed laser experienced by the electron beam can be significantly smoothed when the slippage length is comparable to the pulse length of the seed laser in the modulator. This smoothing effect allows one to create a very uniform bunching distribution and preserve the excellent temporal coherence of seeded FELs in the presence of large phase distortions in the seed lasers.

In this paper, we report the first successful demonstration of using the slippage boosted spectral cleaning technique in a seeded FEL to generate fully coherent pulses from a seed laser with very large phase errors. The results suggest the possibility of generating fully coherent radiation pulse via harmonic up-conversion schemes with the assistance of the proposed technique.

## Results

### Experimental setup

**Figure 1:**
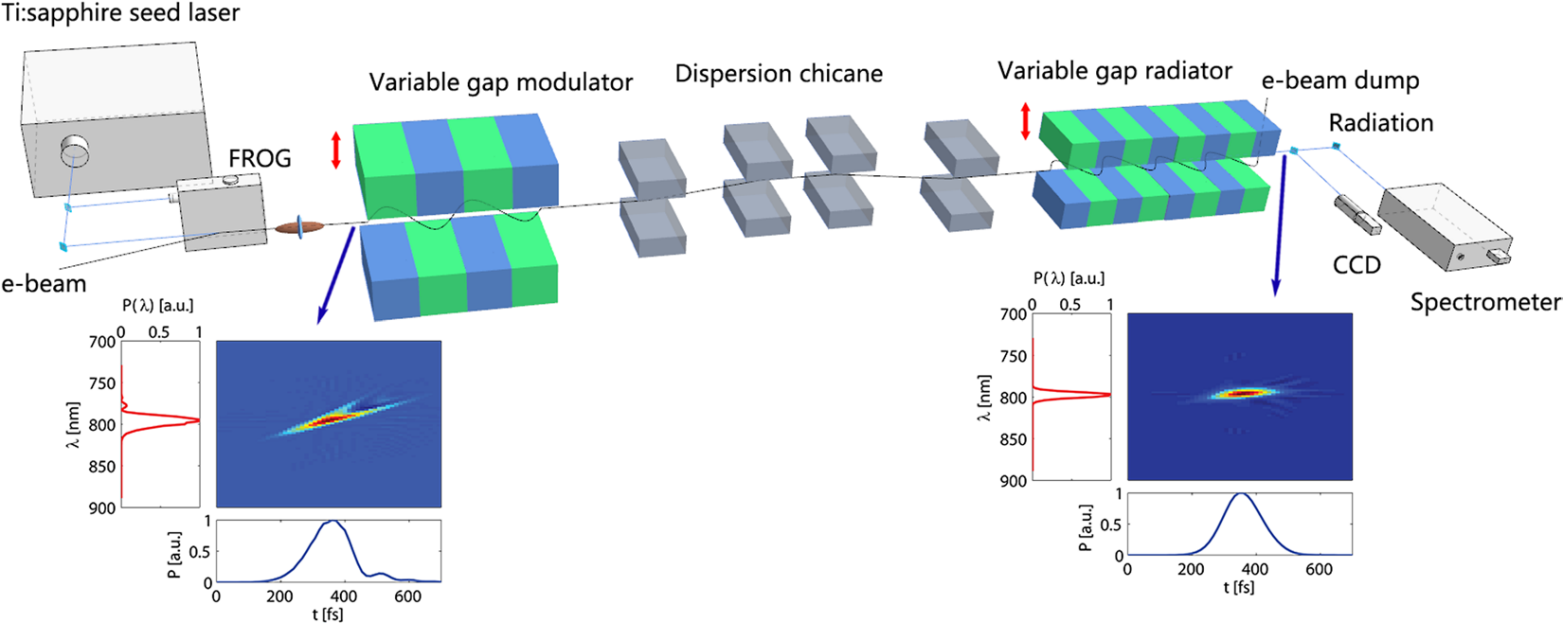
Schematic layout of the experimental setup for testing the slippage boosted spectrum cleaning technique. A seed laser pulse with spectral phase errors (**a**) has been injected into the variable gap modulator to imprint an energy modulation on the electron beam. The energy modulation is converted into density modulation by the dispersion chicane. The pre-bunched electron beam is sent into the variable gap radiator to generate coherent radiation at fundamental and harmonics of the seed which is detected by the downstream CCD and spectrometer. The phase error can be eliminated (**b**) by using sub-harmonic modulation.

The experiment setup is depicted in Fig. 1, where a CHG scheme is adopted for generating coherent radiation. A 148 MeV electron beam with bunch charge of 200 pC, pulse duration of about 8.8 ps co-propagates with an 800 nm seed laser pulse in a variable gap modulator with period length of 5 cm and period number of 10. By tuning the magnet gap of the modulator, the undulator parameter *K* can be easily tuned from 0.8 to 5.6, corresponding to a resonant wavelength from 400 nm to 5 μm. The energy modulation is then converted into density modulation by the following magnetic chicane. After that, the electron beam is sent into the downstream variable gap radiator to generate coherent radiation at fundamental and harmonics of the seed. The radiation properties can be detected with a charge-coupled device (CCD) and a spectrometer (see Methods).

To show the principle of the slippage boosted spectrum cleaning technique, three-dimensional simulations have been performed with the GENESIS numerical code^35^ based on the parameters used in the experiment. To show the superior performance of the proposed technique, a seed laser pulse with second and third order spectral phase errors is utilized in the simulation. The pulse duration of the seed laser is chosen to be 130 fs (full-width at half-maximum, FWHM), which is comparable with the slippage length of the modulator resonant at 4 μm (5^th^ sub-harmonic of the seed). The Wigner distribution function^36,37^ was introduced to fully characterize the longitudinal properties of the laser pulse,

1 $$W(t,\omega ,s)=\int E(t-\tau /2,s){E}^{\ast }(t+\tau /2,s){e}^{-i\omega \tau }d\tau ,$$

where * denotes the complex conjugate, $\omega$ is the laser frequency and $E(t,s)$ is the laser electric field. The Wigner distribution of the seed laser pulse together with the longitudinal profile and spectrum are illustrated in Fig. 1(a), where one can clearly see some high order phase errors. The seed laser interacts with a longitudinal uniform electron beam in the modulator. According to the basic theory of the undulator radiation, after electrons travel one undulator period, the seed laser overtakes the electrons by one resonant wavelength $m{\lambda }_{s}$. As a result, the influence of the seed laser phase to the distribution of the microbunching should be integrated over the slippage length ${L}_{s}=Nm{\lambda }_{s}$, where $N$ is the period number of the modulator. When the slippage length is comparable with the seed laser pulse length (m = 5), the seed laser imperfection experienced by the electron beam can be smoothed and results in a uniform region in the bunching distribution. The radiation pulse emitted from this electron beam will have a similar pulse duration (137 fs, FWHM) with the seed laser but a nearly flat phase, as shown in Fig. 1 (b). The spectral chirp only appears in the lateral parts of the radiation pulse, which has negligible effects on the spectral bandwidth. For comparison purpose, we also performed the simulations for a normal modulator (m = 1). It is found from the simulation results that the pulse duration is also about 135 fs, which means that the sub-harmonic modulator will not significantly increase the radiation pulse duration when the slippage length is comparable with the seed laser pulse length because only the central part of the modulation is optimized for FEL generation. This phenomenon has also been observed in other simulations in ref.^33^

### Preparation of the seed laser with significant phase errors

**Figure 2:**
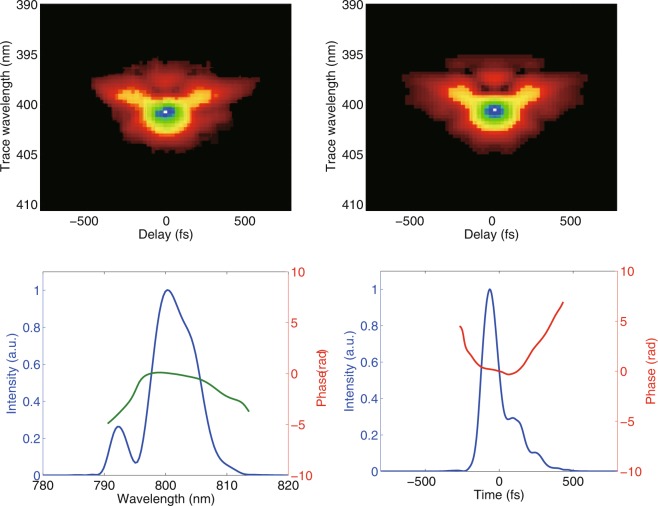
Longitudinal properties of the seed laser pulse measured with FROG. Experimental (**a**) and retrieved (**b**) FROG traces. Retrieved intensity and phase in the temporal (**c**) and spectral (**d**) domains.

In our experiment, the seed laser pulse is produced by a chirp-pulse-amplification titanium-sapphire laser system at 800 nm. The initial pulse length (close to Fourier-transform limit) is about 83 fs (FWHM), which is the normal operating condition for our facility. In order to clearly show the spectral cleaning effect, high order dispersions have been induced by tuning the position and angle of the second grating in the compressor of the amplifier. Pulse characterization of the seed laser was performed with the second-harmonic generation frequency-resolved optical gating (SHG FROG) method^38^. The measurement results are summarized in Fig. 2. The residual second order dispersion introduced a linear chirp into the seed laser and stretched the laser pulse length from 83 fs to about 124 fs. The third order dispersion causes an asymmetric delay of the pulse, resulting in parasite pulses in the temporal domain and sidebands in the spectral domain. With an ideal electron beam and suitable energy modulation amplitude, these large phase errors will be amplified by the harmonic up-conversion process and eventually destroy the longitudinal coherence at very high harmonics.

### FEL measurement results

**Figure 3:**
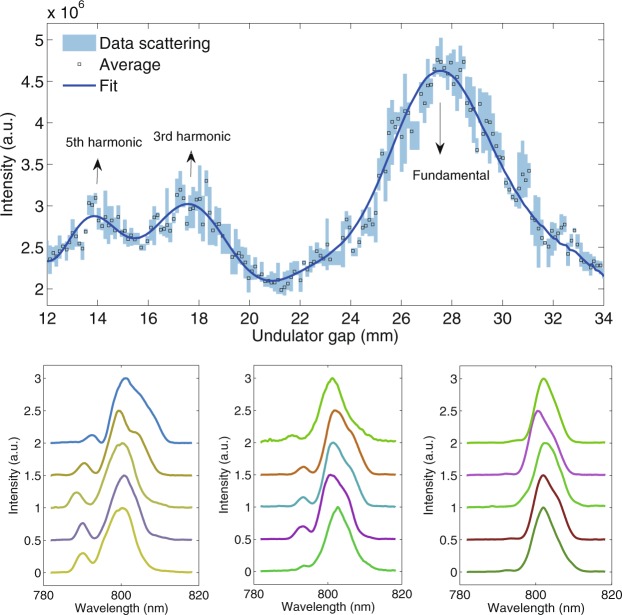
Spectrum cleaning of the fundamental radiation pulse with the slippage boosted effect in the modulator. (**a**) CHG output intensity as function of the magnetic gap of the modulator. (**b**–**d**) CHG spectra at fundamental wavelength for different resonant wavelengths of the modulator: 800 nm, 2.4 μm and 4 μm, respectively.

In the experiment, a transform limit 800 nm laser pulse (83 fs) was first adopted and the resonant wavelengths of both the modulator and radiator were tuned to the fundamental wavelength of the seed laser. A CCD camera is utilized downstream of the radiator to detect the transverse spot and intensity of the harmonic radiation. The radiation properties in frequency domain can be investigated with a spectrometer (see Methods). Energy modulation was achieved when the electron beam and laser beam overlap spatially and temporally in the modulator (see Methods). Various parameters such as the timing of the seed laser, the gaps of the undulators and the strength of the dispersion had been optimized to maximize the radiation power. Then the seed laser power has been optimized according to the spectrum of the radiation to avoid electron overbunching and radiation pulse splitting^22,39^. After that, the seed laser has been stretched to 124 fs to induce phase errors. The output spectra for five typical shots of CHG at fundamental wavelength (800 nm) are shown in Fig. 3(b). It is clearly shown that the spectra of CHG directly inherit the properties of the seed laser with similar bandwidth and apparent spectral sidebands. By changing the gap of the modulator gradually while keeping the resonance of the radiator at 800 nm, the output intensity of CHG is recorded as shown in Fig. 3(a). Three peaks appeared sequentially when the gap is tuned from 12 mm to 34 mm, corresponding to the resonant wavelengths of 800 nm (fundamental), 2.4 μm (3^rd^ sub-harmonic) and 4 μm (5^th^ sub-harmonic), respectively. Compared with the modulation at fundamental wavelength, the peak values of the output pulse energy are reduced by about 2–3 times for sub-harmonic modulations. Figure 3(c,d) show the typical CHG spectra for the sub-harmonic modulations, where the seed laser power had been increased for sub-harmonic modulations to get similar radiation power as the fundamental modulation. The spectral sidebands are significantly reduced in Fig. 3(c) and nearly disappeared in Fig. 3(d). These measurement results coincide with theoretical predictions and clearly show the spectral cleaning effect.

**Figure 4:**
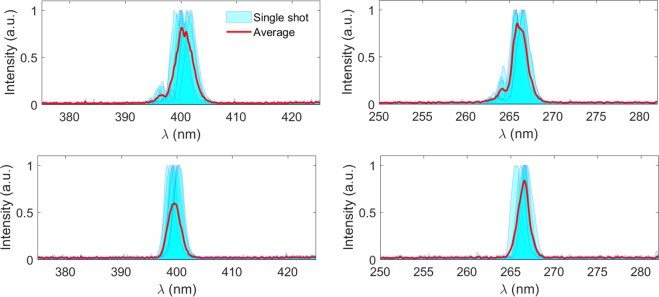
Comparison of output spectra at different harmonics of the seed. 2^nd^ and 3^rd^ harmonic radiation spectra for the modulator resonant at 800 nm (**a**,**b**) and 4 μm (**c**,**d**).

**Figure 5:**
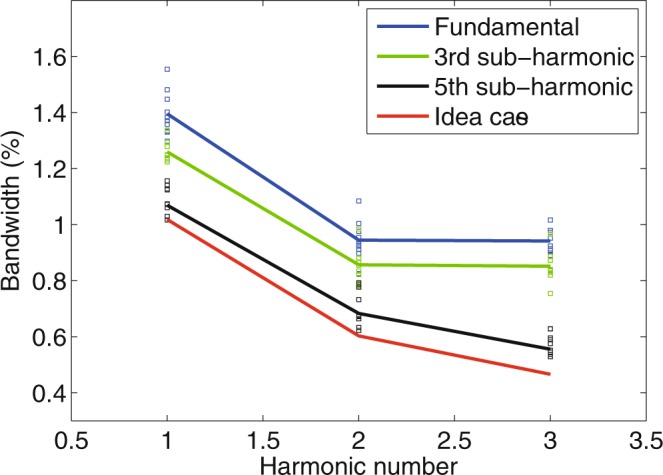
Comparison of output spectral bandwidths (FWHM) from experiment and simulations for different optimized conditions. The modulator is resonant at 800 nm (blue square) 2.4 μm (green square) and 4 μm (black square). Simulations are performed with an ideal Gaussian seed laser pulse (red line).

By tuning the gap of the radiator, we also measured the spectra of the coherent harmonic radiation at second (400 nm) and third (266 nm) harmonics of the seed. Figure 4 compares the measurement spectra for 2^nd^ and 3^rd^ harmonic radiation with the modulator tuned at the fundamental wavelength (800 nm) and 5^th^ sub harmonic (4 μm) of the seed laser. One can find the similar cleaning effects that eliminated the sidebands in the spectra. Figure 5 summarizes the measurement spectral bandwidths for various harmonics together with simulation predictions. When the modulator resonates at 800 nm, the average value of the output bandwidth at fundamental wavelength is about 1.41% (FWHM), which is close to that of the seed laser. The output bandwidth is reduced to about 1.26% and 1.06% for sub-harmonic modulations at 2.4 μm and 4 μm. For higher harmonic radiation, the output bandwidth of CHG will be broadened mainly due to two effects: the natural pulse shortening^16,21,39,40^ and the phase error multiplication. To make things clear, simulations were performed with experiment parameters but an ideal transform-limit Gaussian pulse as the seed laser. The pulse duration of the seed laser is also set to be 124 fs, and the modulator is resonant at the fundamental wavelength of 800 nm. For a 124 fs transform limited Gaussian pulse with central wavelength at 800 nm, 400 nm and 266 nm, the bandwidth should be about 0.96%, 0.47% and 0.32%, respectively. However, due to the pulse shortening effect, the corresponding spectral bandwidths are broadened to be about 1%, 0.6% and 0.46% in the simulations. Excluding the pulse shortening effect, the bandwidth broadening for high harmonic radiation is mainly cause by phase error amplification. When the slippage length is shorter than the seed laser pulse length, i.e. the modulator resonates at 800 nm or 2.4 μm, the phase errors induced by the seed laser cannot be fully eliminated, and the radiation bandwidths are much wider than the ideal case, as shown in Fig. 5. The deviation between the experiment and simulation results becomes larger for higher harmonics due to the phase error multiplication. The spectral cleaning effect becomes significant when the modulator resonates at 4 μm. Under this condition, the average spectral bandwidths at 400 nm and 266 nm are measured to be about 0.67% and 0.55%, which are quite close to those of the ideal case. These results demonstrate the effectiveness of the slippage boosting effect for spectrum cleaning and generating nearly transform limited radiation pulses at high harmonics.

## Discussion

We have demonstrated the spectral cleaning method in a seeded FEL based on the slippage boosted effect in the modulator. It is found that, by adopting a sub-harmonic modulator to enhance the slippage length to a comparable level of the seed laser pulse length, the initial large phase errors can be significantly smoothed and the production of nearly transform-limited radiation pulses is possible. 3D Simulations have been performed and the results are consistent with the experiments. It’s worth stressing here that, limited by the harmonic number that can be achieved at the SDUV-FEL, we enlarge the phase errors in the seed laser to show the efficiency of the proposed method. The practicability of the proposed method depends on the application of it onto an x-ray FEL operated at very high harmonics with a seed laser with limited phase noises. Soft x-ray seeded FELs usually start from ultraviolet seed lasers at ~260 nm. In this case, a longer modulator with 30 periods and resonating at 5^th^ sub-harmonic (1330 nm) will be sufficient to eliminate the phase error induced by the seed laser with pulse duration of about 100 fs. The slippage boosted spectral cleaning method paves the way towards fully coherent x-ray generation with external seeded FEL schemes and provides a novel method for precisely controlling the temporal phase of ultra-short laser pulses.

## Methods

### Machine setup

Experiments were conducted at the Shanghai Deep Ultraviolet FEL facility (SDUV-FEL), which is a multipurpose test facility for FEL principle studies. The linear accelerator (LINAC) consists of an S-band photo-injector, four S-band accelerating modules and a magnetic chicane as the bunch compressor. In the experiment, the LINAC provided an electron beam with central energy of 148 MeV, bunch charge of 200 pC, normalized emittance less than 4 μmrad, project energy spread of about 0.2 MeV and pulse duration of about 8.8 ps (FWHM). The bunch compressor had been turned off during the experiment to minimize the possible effects from the nonlinear energy curvature on the final CHG spectrum. The seed laser came from a commercial COHERENT titanium-sapphire laser system, which can provide up to 3 micro-Joules energy with 83 fs (FWHM) pulse duration. The laser pulse from amplifier was split in two branches: one pulse with energy of about 1 μJ was sent into a FROG (GRENOUILLE: 8–50-ECO, Swamp Optics) to detect the longitudinal properties of the pulse on-line; The other pulse was delivered by a 20 meters long optical transport system for the seeding purpose. The seed laser pulse energy could be continually tuned from 0 to 200 μJ at the entrance of the modulator. The undulator system consists of two stages of seeded FELs. In this experiment, we only used one permanent magnet modulator (50 mm × 10), one electromagnetic chicane and one segment of the permanent magnet radiator (40 mm × 40). The magnet gap of the modulator was scanned from 12 mm to 40 mm to optimize the laser-electron beam interaction at different wavelengths. The magnet gap of the radiator was tuned to 16.4 mm, 23.2 mm and 35.0 mm to fulfill the resonant condition for the harmonic radiation at 800 nm, 400 nm and 266 nm, respectively. The momentum compaction R_56_ of the chicane can be continually tuned from 0 to 10 mm and had been fixed to be about 4 mm during the experiment. The CCD camera and the spectrometer were placed close to an in-vacuum reflecting mirror downstream of the radiator. The intensity variation of the radiation pulse as a function of the modulator gap was measured by the CCD camera. The spectrometer is a commercial one (TRIAX-550, Jobin Yvon) with a focal length of 550 mm, spectral coverage from 150 nm to 1500 nm, and could provide a resolution better than 0.1 nm.

### Spatial and temporal overlap

By using Yttrium aluminum garnet screens before and after the modulator, the spatial overlap can be easily achieved by placing both the electron beam and laser beam on the same position. The transverse size of the laser beam spot was tuned to be at least 5 times larger than the electron beam for reducing the FEL output fluctuations caused by pointing jitters. The temporal overlap had been established through two steps: first, initial coarse temporal overlap with an accuracy of 30 ps was achieved by detecting signals of the seed laser pulse and the spontaneous emission from the modulator simultaneously by a fast photodiode (2 GHz). After that, the exact temporal overlap was obtained by tuning the delay line of the seed laser until we observe the coherent radiation signal on the CCD camera.
